# The usefulness of ultrasonography as a dynamic measurement system for visualizing root canal working length: an in vivo study

**DOI:** 10.1186/s12903-024-04562-6

**Published:** 2024-07-16

**Authors:** İrem Eren, Yeşim Deniz

**Affiliations:** 1 Dentistry Faculty, Department of Endodontics, Çanakkale Onsekiz Mart University, Çanakkale, Turkey; 2 Dentistry Faculty, Department of Dentomaxillofacial Radiology, Çanakkale Onsekiz Mart University, Sahil Yolu Street, No:5, Çanakkale, 17100 Turkey; 3 Graduate School of Dentistry, Department of Oral and Maxillofacial Radiology, Osaka University, Suita, Osaka Japan

**Keywords:** Tooth apex, Ultrasonography, Root canal preparation

## Abstract

**Objective:**

Although apex locators are generally effective tools for determining root canal working length, they may produce inaccurate results in some cases. The present study aimed to evaluate the efficacy of ultrasonography as an alternative method for measuring root canal length.

**Materials and methods:**

Forty-seven anterior teeth with apical lesions were selected for the study. Initially, an electronic apex locator was used to measure the working length. Subsequently, ultrasonography was employed to visualize the root apex and determine the working length. During ultrasound imaging, a K-file No. 15 was inserted into the root canal until its tip was visible on the ultrasound monitor. Measurements obtained from both methods were compared using an independent sample t-test. Correlations were assessed with the Pearson correlation coefficient, and agreement was determined using the Bland‒Altman plot.

**Results:**

The mean working canal length was 19.9 mm for the apex locator and 20.6 mm for the ultrasonography-guided method. No significant differences were observed between the data obtained using the apex locator method and the data obtained using the ultrasonography guidance method. Furthermore, a high level of agreement was identified between the two techniques.

**Conclusion:**

Ultrasonography can be used to visualize the apex effectively and determine canal length, especially when canal length determination is uncertain for various reasons.

**Supplementary Information:**

The online version contains supplementary material available at 10.1186/s12903-024-04562-6.

## Introduction

Successful root canal treatment primarily requires the determination of the working length of the root canal. Inaccurate determination can lead to various complications, such as apical perforation, improper cleaning and shaping or over/underfilling of root canals [1]. Methods such as tactile sensation, radiographic techniques, and electronic apex locators (EALs) have been studied for their reliability in determining root canal length [2, 3]. However, tactile sensation alone is insufficient for determining the actual working length [4]. To date, EALs and periapical radiographs—both separately and in combination—have been useful in determining working length. Additionally, anatomical averages and knowledge of anatomy and moisture at paper points are traditional methods used during root canal treatment. However, these methods have limitations and are not definitive in localizing the apical constriction [5]. The use of periapical radiographs, the only method allowing visual inspection, cannot consistently determine working length due to anatomical variations between teeth and superimpositions caused by the nature of 2D geometry [6]. EALs eliminate most of the problems associated with radiographic measurements. They are more accurate, easier, and faster. They also eliminate limitations caused by the harmful effects of X-rays [5]. However, this technique requires special devices, and its accuracy is affected by the electrical condition of the root canal. The presence of tissue and conductive irrigants in the canal changes the electrical properties and leads to measurement errors [3].

Unlike intraoral radiography, ultrasonography (US) is primarily used to examine soft tissues. However, the root structure behind the cortical bone cannot be visualized by US because ultrasound cannot pass through hard tissues such as the buccal cortical bone. In patients with cortical bone resorption in the periapical region due to the inflammatory process, it may be possible to visualize the periapical tissues clearly in real time [7, 8]. Few studies have shown the possibility of tracing beyond the cortical bone in the presence of a periapical lesion without complete cortical resorption.

 [9, 10]. Some studies have demonstrated the potential use of US in imaging periapical lesions and evaluating the blood supply to the periapical region with color Doppler [7, 8, 11]. On the other hand,

there are no studies showing that it can be used for real-time imaging of the periapical region during endodontic treatment, such as for determining the working length under in vivo conditions.

The aim of this in vivo study was to evaluate the effectiveness and potential benefits of noninvasive, radiation-free US imaging in determining the working length of the root canal as an alternative to routinely used EAL and periapical radiography methods. Thus, the usability of the US method, as an alternative to EALs, was tested under in vivo conditions.

## Materials and methods

This study was reviewed and approved by the Canakkale Onsekiz Mart University Clinical Research Ethics Committee under ethical approval number 2023/02–15. The study was conducted in accordance with all ethical principles and obligations of the Declaration of Helsinki. After the study was explained, all subjects and/or their legal guardian(s) signed an informed consent form which informed that they allow for publication of identifying information/images in an online open-access publication.

### Sample size and sample selection

A power test was performed for an effect size of 0.5, an alpha error of 0.05 and a test power of 0.90. Although the total sample size was 44, the study included 50 patients to mitigate potential errors. Among them, 25 were female, and 25 were male. Informed consent was obtained from all patients included in the study.

#### Inclusion criteria

The study included teeth with fully developed roots and a single, straight root canal, as classified by Vertucci’s Type I classification [12]. Another inclusion criterion was the presence of chronic apical periodontitis or apical radicular cysts, as evidenced by periapical radiographs.

#### Exclusion criteria

Teeth were excluded if they exhibited root fractures, root resorption, radicular caries, ill-defined periapical radiolucency, or partially or completely blocked root canals. Additionally, teeth with prior endodontic treatment or systemic conditions associated with bony pathology, such as hyperparathyroidism, Paget’s disease, fibrous dysplasia, etc., were excluded from the study.

Prior to beginning endodontic treatment, a periapical radiographic examination was conducted to confirm the inclusion criteria and verify the absence of root resorption or canal curvatures.

### Electronic measurement of root canal length

After radiographic assessment, coronal access was established. Subsequently, debris and any remaining pulp tissue were meticulously removed using a K-file (Dentsply Maillefer, Ballaigues, Switzerland) of appropriate thickness. The procedure was performed under visualization with a surgical microscope (Click OMS2360, Zumax Medical, China) at a magnification of ×20.0.

**Fig. 1:**
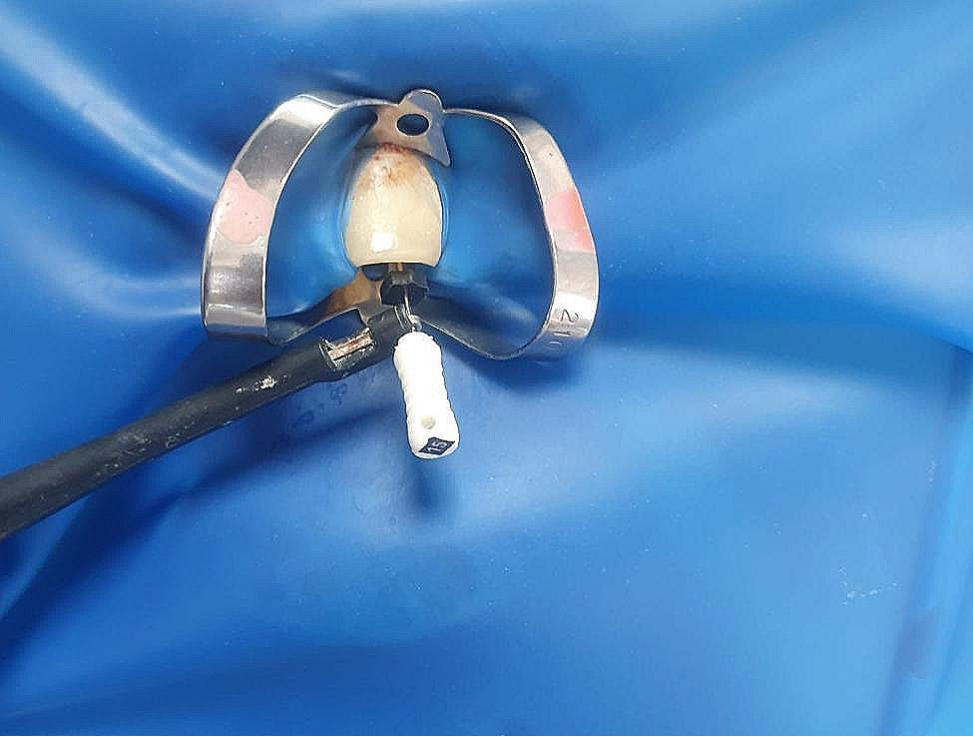
The K-file was inserted into the root canal and the electrode of the EAL was attached to the K-file during EAL measurements

To electronically measure the canal length, a Raypex 6 Apex locator device (VDW, Munich, Germany) was used. Prior to the electronic localization procedure, the canals were irrigated with 1.5 ml of 5.25% sodium hypochlorite (NaOCl) using a 27-gauge needle (Ayset Medical Products, Adana, Türkiye), positioned 3 mm short of their estimated working length. After irrigation, any remaining pulp materials were removed using cotton pellets. One electrode was positioned in contact with the lip, while a K-file was attached to the other electrode for electronic measurement (Fig. 1).

The file was gently introduced into the root canal until the warning red dot was observed and then pulled back until the measurement was repeatable and stable on the third green bar, corresponding to 0.5 mm short of the radiographic apex, in accordance with the manufacturer’s instructions. The K-file was carefully advanced until the warning green bar signal appeared on the LED screen. When the “APEX” signal was observed, the K-file was gently retracted to the localization point indicated by the green bar on the LED screen. Next, the stopper on the K-file was carefully adjusted according to the reference level based on the incisal edge of the teeth. The K-file was slowly withdrawn from the canal, and measurements between the stopper and the file tip were taken using a ruler (Dentsply Maillefer, Ballaigues, Switzerland). The EAL measurements were repeated three times, and the average of the measurements was used as the working length of the root canal.

### Dynamic measurement of root canal length using an ultrasonography device

#### Device and probe specifications

The ultrasonographic (US) examination was performed by a dentomaxillofacial radiologist using a Hitachi Arietta 65 US machine (Hitachi Aloka Medical Systems, Tokyo, Japan) equipped with a hockey probe. The probe was operated within the 3- to 15-Hz frequency range, with an average scanning frequency of 7.5 MHz and a 200-degree field of view. The US device provided an image width of 25 mm, depth of 20 mm, and frame frequency of 45 FPS. The 21.5-in device monitor presented images with the depth of a 12-bit analog-to-digital converter. B-mode dynamic scanning was used to capture longitudinal images of the apical region. Only a single ultrasound examination for canal length measurement was recorded from each patient.

#### Initiation of the US examination

**Fig. 2:**
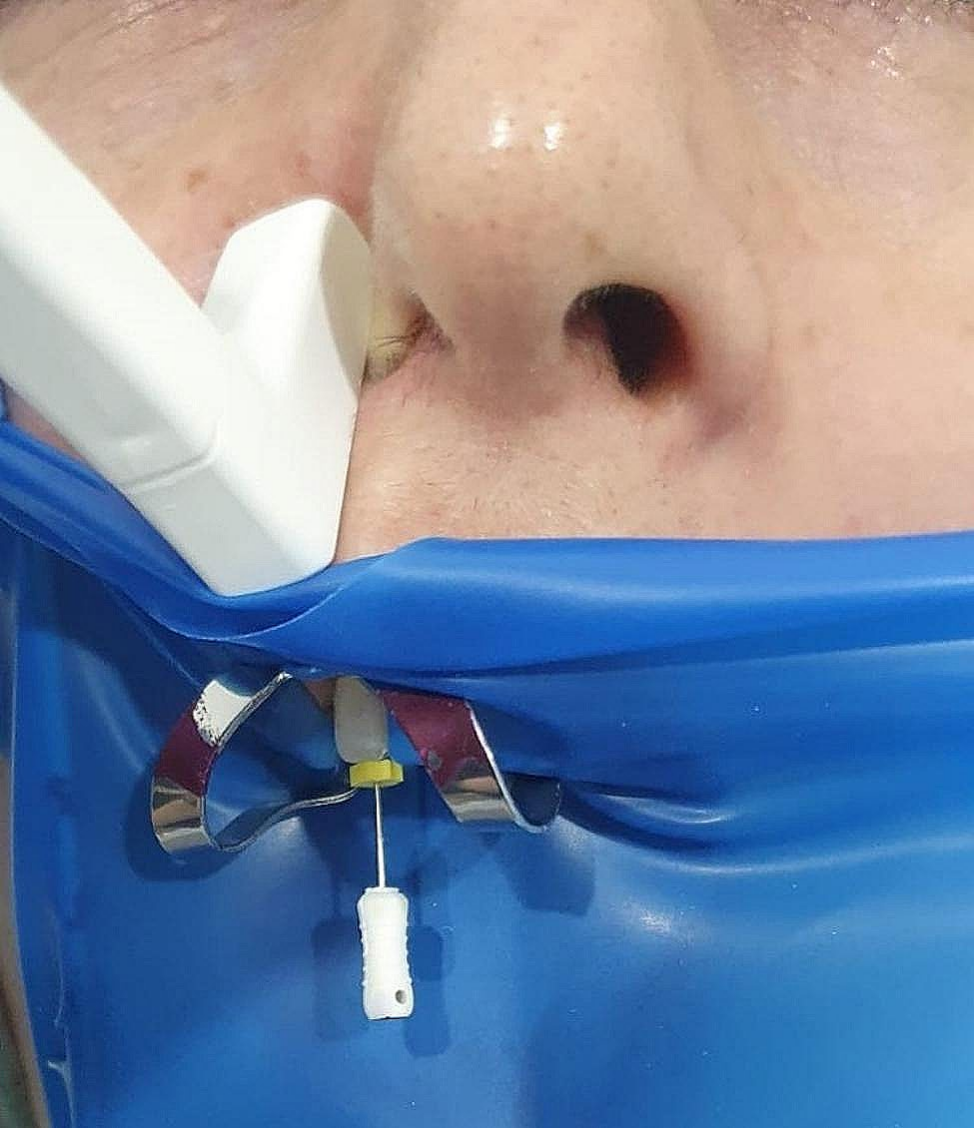
The ultrasound examination of a patient during root canal treatment revealed that the canal file was inserted into the canal. The ultrasound procedure is performed extraorally, with the hockey probe placed longitudinally on the patient’s relevant tooth

First, to measure the working length, the tooth apex was visualized using US. The surface of the US probe was coated with a layer of US gel (Ultragel, Medicon, India) and placed extraorally in contact with the skin over the region corresponding to the area of interest of the tooth root. The hockey probe was then manually positioned longitudinally and parallel to the axes of the teeth (Fig. 2).

#### Determination of anatomical landmarks on US imaging

**Fig. 3:**
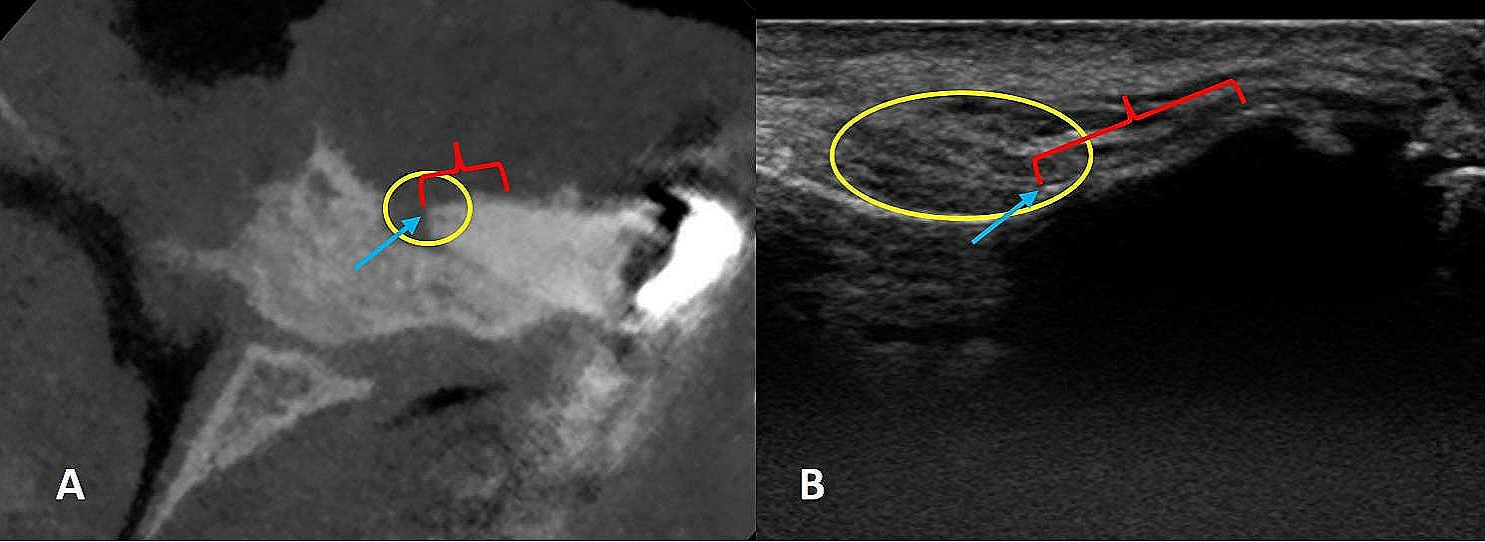
(**A**) CBCT image of a patient included in the study (which was taken for a different reason) shows a periapical lesion related with apex of right santral incisor teeth on cross-sectional view. (**B**) US image of the same tooth. The blue arrows indicate the root apex, the yellow circles denote the periapical region, and the red indicators define the root of the tooth. The CBCT image reveals a periapical lesion and bone resorption. In the US image of the same area, due to bone resorption, the ultrasonic wave has been transmitted the root and reflected back. Hence, the root apex (blue arrows) can be observed as hyperechoic within the apical lesion (yellow circle). Also, due to bone resorption in the relevant area, the ultrasonic wave has been able to reach the apical third of the root and reflected back. Therefore, the line indicated by the red marker appears more hyperechoic, while behind the opaque line, where the ultrasonic wave has not been transmitted beyond, no signal could be obtained, resulting in a anechoic appearance

**Fig. 4:**
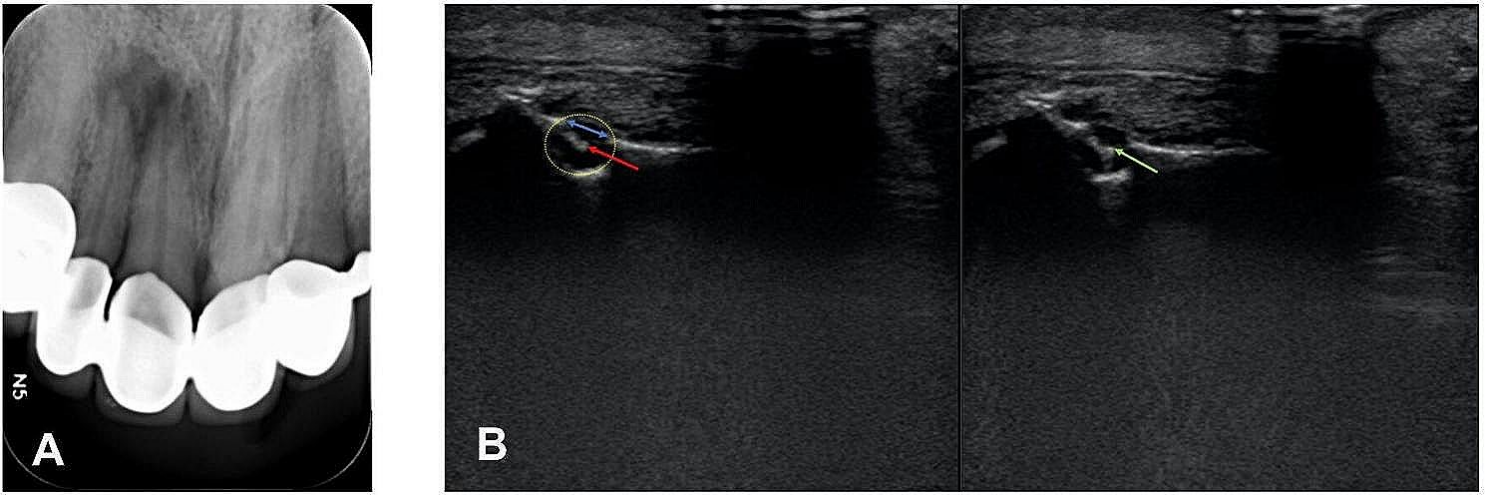
(**A**) A periapical image of a right central and lateral tooth with apical radiolucency. (**B**) Ultrasound image of the same patient, captured in real-time with a canal file inserted into the root canal. In the image, the green line represents the brightly visible tip of the canal file instrument, while the red line marks the root apex, appearing white but darker than the instrument tip. The blue line indicates the region of resorption on the cortical bone corresponding to the teeth

The root apex was assessed to obtain a clear view of the apical region. In the US image, the most hyperechoic structure at the top of the image was identified as the alveolar bone owing to the complete reflection of the US wave. Hyperechoic lines observed in areas with thinned cortical bone were also recognized as representing tooth roots. In addition, apical lesions were identified as slightly hypoechoic areas around the tooth apex (Figs. 3 and 4). As can be seen from Fig. 3, the US image obtained when the probe is placed in the longitudinal position actually corresponds to a cross-sectional image similar to cross-sectional sections in CBCT.

#### Determination of the major apical foramen using a K-file

Real-time US images were obtained by a dentomaxillofacial radiologist who was blinded to the electronic apex locator (EAL) results, and the working canal lengths were determined using a K-file. During the US-based canal length measurement, the K-file was inserted in the root canal until its tip was visible on the US monitor at the level of the major apical foramen (see Supplementary Video). The canal file could be distinguished on the dynamic US image owing to its hyperechoic characteristics. As it exits the major apical foramen, it enters the apical lesion area, which appears hypoechoic. The contrast in imaging characteristics, with the canal file appearing hyperechoic despite the hypoechoic appearance of the infected apical region, aided in its prompt recognition upon exiting the major apical foramen. Figure 3 depicts the US and periapical images of the same patient (Fig. 4).

#### Recording the root canal length

After observing the file tip at the apical foramen, it was retracted by 1 mm within the canal to ensure positioning at the minor apical foramen. Subsequently, the silicone stop on the file was stabilized at the incisal edge of the tooth. Thus, the point where the file disappeared from the US image was fixed with a stopper and the US-guided canal length distance was transferred to the file. Therefore, US imaging was used only to determine the position of the canal instrument at the apical region. No measurements were made directly on the image. The file was then withdrawn, and the distance between the rubber stop and the file tip was measured using the same ruler (Dentsply). This measurement represents the working length of the root canal (Fig. 4). The US measurements were repeated three times, and the average was calculated.

### Statistical analysis

The data collected were statistically analyzed using the SPSS v27.0 software (IBM Corp., Armonk, NY). Descriptive statistics were used to describe the data. All data showed a normal distribution. The differences between the US-guided and EAL measurements were compared using independent sample *t*-tests. The correlation between the two techniques was determined using the Pearson correlation coefficient and Bland‒Altman plot. The level of significance was established at *P* < 0.05.

## Results

**Fig. 5:**
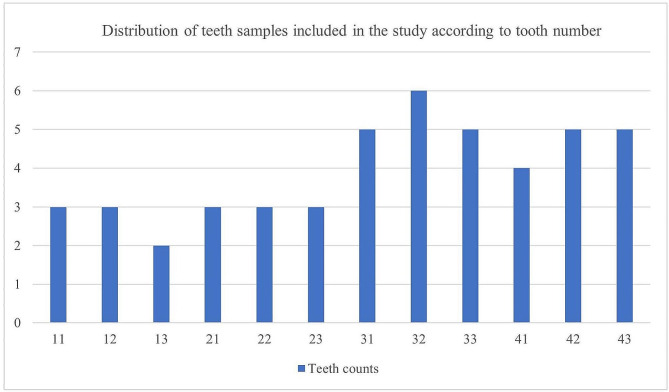
Distribution of teeth included in the study according to the number of teeth

This study included 50 patients (50 anterior teeth) who underwent initial canal length measurement using an apex locator. Of these patients, 25 were female and 25 were male. The patients’ mean age was 42.2 years (range, 20–66 years) for the women and 46.4 years (range, 29–68 years) for the men. Imaging could not be obtained for tooth No. 13 in two patients and tooth No. 43 in one patient. The distribution of the affected tooth among the 47 patients was as follows (Fig. 5): tooth No. 11, 6.38% (*n* = 3); tooth No. 12, 6.38% (*n* = 3); tooth No. 13, 4.25% (*n* = 2); tooth No. 21, 6.38% (*n* = 3); tooth No. 22, 6.38% (*n* = 3); tooth No. 23, 6.38% (*n* = 3); tooth No. 31, 10.63% (*n* = 5); tooth No. 32, 12.76% (*n* = 6); tooth No. 33, 10.63% (*n* = 5); tooth No. 41, 8.51% (*n* = 4); tooth No. 42, 10.63% (*n* = 5); and tooth No. 43, 10.63% (*n* = 5).

The mean working canal lengths, as recorded by the apex locator and US-guided method, were 19.9 and 20.6 mm, respectively (Table 1). The canal working length measurements obtained using the apex locator were not significantly different from the working length ranges obtained under real-time US guidance. This result was confirmed through an independent *t*-test, which yielded a *P* value of 0.980 (Table 1).

**Table 1:** The results of mean root canal working lengths as recorded by the apex locator and the US guided method

	*N*	Mean		Standard Deviation	Independent sample t test *P* value	Pearson Correlation	Correlation *p* value
EAL	47	19.99		2.96	**0.980**	**0.894**	< 0.001
US	47	20.60		2.88			

Bold fonts: no significant differences according to independent t-test and Pearson correlation coefficient analysis results

**Fig. 6:**
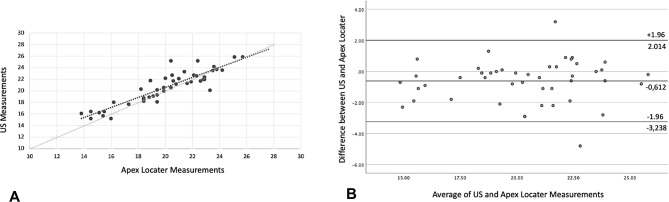
Correlation between the US and the Apex Locator: (**A**) A significant inverse correlation is observed between the US and the Apex Locator. (**B**) The Bland‒Altman plot illustrates strong agreement, with points clustered closely around the mean bias line. Based on our results, these two methods can be used interchangeably

Moreover, when evaluating the agreement between the apex locator and US real-time canal length measurements, we identified a high agreement, with a Pearson correlation coefficient of 0.894 and *P* value of < 0.001 (Table 1; Fig. 6). These indicate similar results between the US-guided real-time and apex locator techniques. Furthermore, in the subsequent analyses, a Bland‒Altman scatter plot analysis was performed to further examine the results (Fig. 6).

## Discussion

Radiographs play a crucial and integral role in endodontic treatment [13]. Radiography uses ionizing radiation; thus, to minimize the potential risks associated with ionizing radiation exposure, it should be performed only when necessary [14]. In the field of endodontic treatments, the development and production of EALs have allowed for the reduction of nonionizing radiation [15]. These devices electronically detect the canal terminus and measure the working canal length on the basis of the principle that the electrical conductivity of the tissues surrounding the root tip is greater than that within the root canal system. When the canal file reaches the periodontal membrane via the apical foramen at the tip of the endodontic instrument, constant electrical resistance is established between the instrument and the oral mucosa. Building upon this fundamental principle, these resistance-based devices can detect periodontal tissue at the apical foramen [16].

Unlike traditional radiographic methods, apex locators operate without ionizing radiation, reducing patient and clinician exposures to harmful rays. In addition, they eliminate the discomfort associated with undergoing multiple radiographic exposures. Furthermore, numerous studies have reported additional advantages of apex locators, such as their precise detection of apical constriction and the major apical foramen [17–19]. In a previous study, apical constriction was calculated using nine different EAL devices. Subsequently, the EAL data obtained were compared with the measurements obtained using micro-CT cross-sectional imaging, which is considered the gold standard for visualizing hard tissues. Connert et al. reported no significant difference in the accuracy of determining the apical constriction or foramen between the nine different apex locators within tolerances of ± 0.5 and 1 mm [19]. The device, which is a previous version of that used in the present study, was evaluated for its efficacy in the studies of Connert et al. The accuracy of the device has been reported to be high compared to the values obtained using micro-CT [19].

However, the use of apex locators is associated with several disadvantages. For instance, the presence of metallic restorations, crowns, or posts within the oral cavity can potentially interfere with the accuracy of apex locator measurements. Owing to the use of electricity, to obtain more accurate results, the canal must be either dry or filled with a nonconductive liquid. Excessive salivation may also affect the accuracy of the device. Moreover, the reliance on battery power and the need for periodic calibration for accurate measurements can lead to additional maintenance requirements. Furthermore, apex locators may have limitations in cases involving extremely calcified canals or unconventional canal anatomies, which reduce their applicability. In addition, the presence of peri radicular lesions, incomplete root development in teeth, wide apical foramens, inability of the file used for measurement to match the canal diameter, and blockage of the apical foramens can also lead to a loss of accuracy [20].

In such situations, alternative methods for measuring root canal length and/or enabling non-ionization verification of the measured length may be necessary. This is particularly significant, as conventional radiographic techniques rely on radiation. Therefore, our study aimed to evaluate the viability of using US as an alternative method. US offers real-time visualization and uses a nonionizing technique, which has the potential to improve root canal length measurements without the disadvantages associated with radiation-based approaches.

US, also known as echography, is a safe imaging technique used in various fields of medicine, including dentistry, without reports of harmful effects on humans. Real-time imaging can be achieved using US. In terms of the operational principle of US, ultrasound waves are directed toward the biological tissues of the human body using a probe, and the ultrasound waves reflected from the tissues are collected using the same probe. Depending on the mechanical and acoustic properties of the tissues examined, echoes of different intensities are generated and then transformed into images on a computer screen [21]. The first examination of tooth apical tissues using US was performed by Cotti et al. Researchers have used US to diagnose apical periodontitis and distinguish apical cystic lesions from granulomas [22, 23]. Furthermore, studies have also assessed the healing of apical periodontitis after orthograde [24] and surgical endodontic treatments [25]. In addition, studies have demonstrated the potential of usefulness of US for both imaging periapical lesions and assessing the blood supply to the periapical region using color Doppler [7, 8, 11].

In this preliminary study, US was used to measure the root canal working length in real time by visualizing the root apex under in vivo conditions. Considering that the cortical bone is completely resorbed, an in vitro study by Irem et al. revealed that US was used for real-time imaging of the periapical region during endodontic treatment to determine the working length [26]. EAL and US measurements were performed on teeth embedded in alginate. During the US measurement process, a window was created at the apical region to simulate cortical bone resorption. The study reported that the US provided measurements similar to those of EALs. Our study is the first to measure canal length in an in vivo environment using US, with the significant advantage of real-time imaging. While planning this study, our primary concern in using US to evaluate root apex pathologies was the presence of cortical and trabecular bones, which have the potential to limit the ability of ultrasound waves to access the tooth root apex. For the penetration of ultrasound waves into the apex, studies have indicated that thinning of the cortical thickness may suffice [7, 11, 27]. The reason for including teeth with apical radiolucency is that this examination is a pioneering study that aimed to ensure the observation of the tip of the canal file as it extends beyond the minor apical foramen. The absence of apical radiolucency make it challenging to differentiate the file tip from other anatomical structures. When comparing US data with histopathological findings, we found that US exhibited high diagnostic accuracy. US images were successfully obtained in cases involving thin anterior buccal bones. In our research, extraoral imaging was performed because of the potential hindrance of the intraoral probe by the structure of the vestibular sulcus, similar to the approach used in a previous study [27]. Furthermore, reports have highlighted the ability of US to assess periapical pathologies in potentially thinned cortical bone in the anterior teeth. In a comparison of diagnostic success rates, the highest sensitivity and specificity were observed with US at 95.2%, followed by digital radiography at 55.6% and conventional radiography at 47.6% [28]. In another study that involved bovine mandibular bone, intraosseous lesions could be visualized with a high degree of accuracy and reliability regardless of cortical bone thickness. US demonstrated a remarkable sensitivity of 97.3% and a negative predictive value of 89% [29]. In addition, US could be used to detect intraosseous lesions by establishing a cortical thickness threshold of 1.1 mm for the examination of intrabony defects [30]. We believe that the inclusion of anterior teeth with apical granulomas, apical cysts, and radiolucent lesions in our study inevitably led to bone thinning in cases of this nature. This outcome was a natural consequence of the specific conditions. The thinner bone in the anterior region further contributed to this effect. Consequently, images were obtained for 47 of the 50 teeth in the present study. Throughout the present study, a challenge was encountered in obtaining images from three patients. This was associated with the increased thickness and/or density of the cortical bone, which might have obstructed the imaging process.

The primary limitation of this study is that it was conducted without knowledge of cortical bone thickness. To obtain certain cortical bone information, CBCT imaging would have been necessary. However, to avoid radiation exposure, we preferred conducting our study with precise cortical bone thickness data. Another limitation of the present study was that cases with apical lesions were included to visualize the extent of the endodontic file beyond the apical foramen. In addition, owing to the pioneering nature of this study, we focused our examination solely on the anterior teeth. We plan to extend our research in a future study to encompass the molar and premolar regions, particularly in patients with combined periodontal-endodontal lesions, as periodontal bone loss can allow the US wave to pass through the apical region. In future studies, we will aim to specifically investigate cases where EALs cannot be used to compare tooth data in different positions and assess measurements made by individuals with varying levels of experience.

## Conclusion

In conclusion, consistent measurements were achieved using both US and EAL, with a high correlation between the US and EAL data. US offers a nonradiation technique and real-time operation similar to that of EAL. However, a main advantage of using US is its ability to visualize the apical region. When providing accurate information about the major apical foramen could not be ensured, US can be used to visualize the apex, particularly in patients with thinned cortical bone and apical lesions. It can provide visual information that supports EAL, especially in cases involving teeth with metallic restorations, crowns, and posts; excessive salivation; or instances where the canal length obtained for any reason is uncertain.

### Electronic supplementary material

Below is the link to the electronic supplementary material.

Supplementary Material 1

Supplementary Material 2

Supplementary Material 3

Supplementary Material 4

Supplementary Material 5

Supplementary Material 6

Supplementary Material 7

Supplementary Material 8

Supplementary Material 9

## Data Availability

The data that support the findings of this study are available upon request from the corresponding author.
