# Supplementary material for: The usefulness of ultrasonography as a dynamic measurement system for visualizing root canal working length: an in vivo study
Source: BMC Oral Health. 2024 Jul 16;24:801. doi: 10.1186/s12903-024-04562-6 (PMC11251373; doi:10.1186/s12903-024-04562-6)
Supplement: Supplementary file 2 — Supplementary Material 2 [file 12903_2024_4562_MOESM2_ESM.docx]

**PRILE 2021 Flowchart**

***From: Nagendrababu V, Murray PE, Ordinola-Zapata R, Peters OA, Rôças IN, Siqueira JF Jr, Priya E, Jayaraman J, Pulikkotil SJ, Camilleri J, Boutsioukis C, Rossi-Fedele G, Dummer PMH (2021) PRILE 2021 guidelines for reporting laboratory studies in Endodontology: a consensus-based development. *International Endodontic Journal* May 3. doi: 10.1111/iej.13542.** [**https://onlinelibrary.wiley.com/doi/abs/10.1111/iej.13542**](https://onlinelibrary.wiley.com/doi/abs/10.1111/iej.13542)**.**

**For further details visit: http://pride-endodonticguidelines.org/prile**
