# Supplementary figures and images for: The usefulness of ultrasonography as a dynamic measurement system for visualizing root canal working length: an in vivo study
Source: BMC Oral Health. 2024 Jul 16;24:801. doi: 10.1186/s12903-024-04562-6 (PMC11251373; doi:10.1186/s12903-024-04562-6)

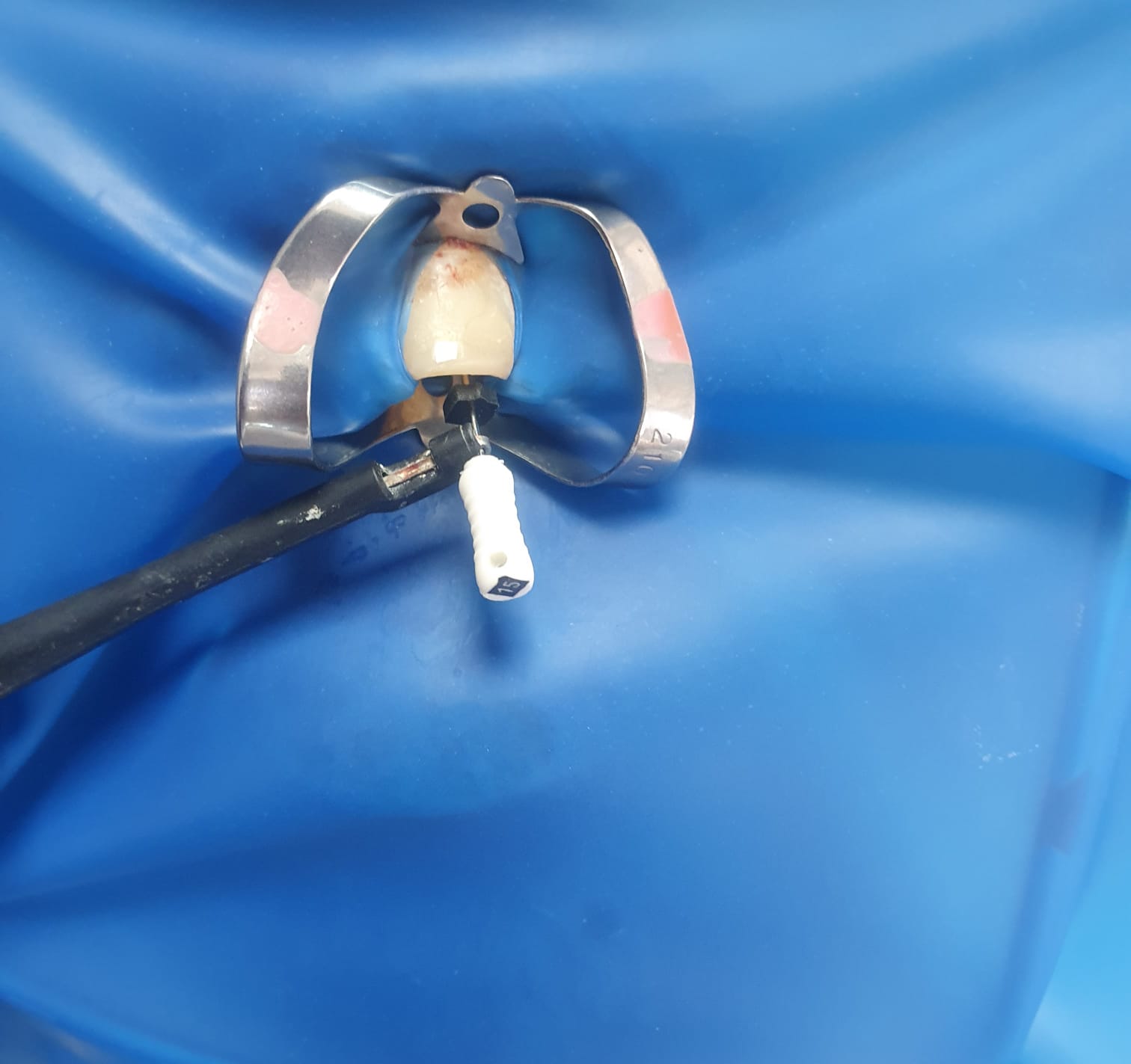

Supplement: Supplementary file 3 — Supplementary Material 3 [file 12903_2024_4562_MOESM3_ESM.jpeg]

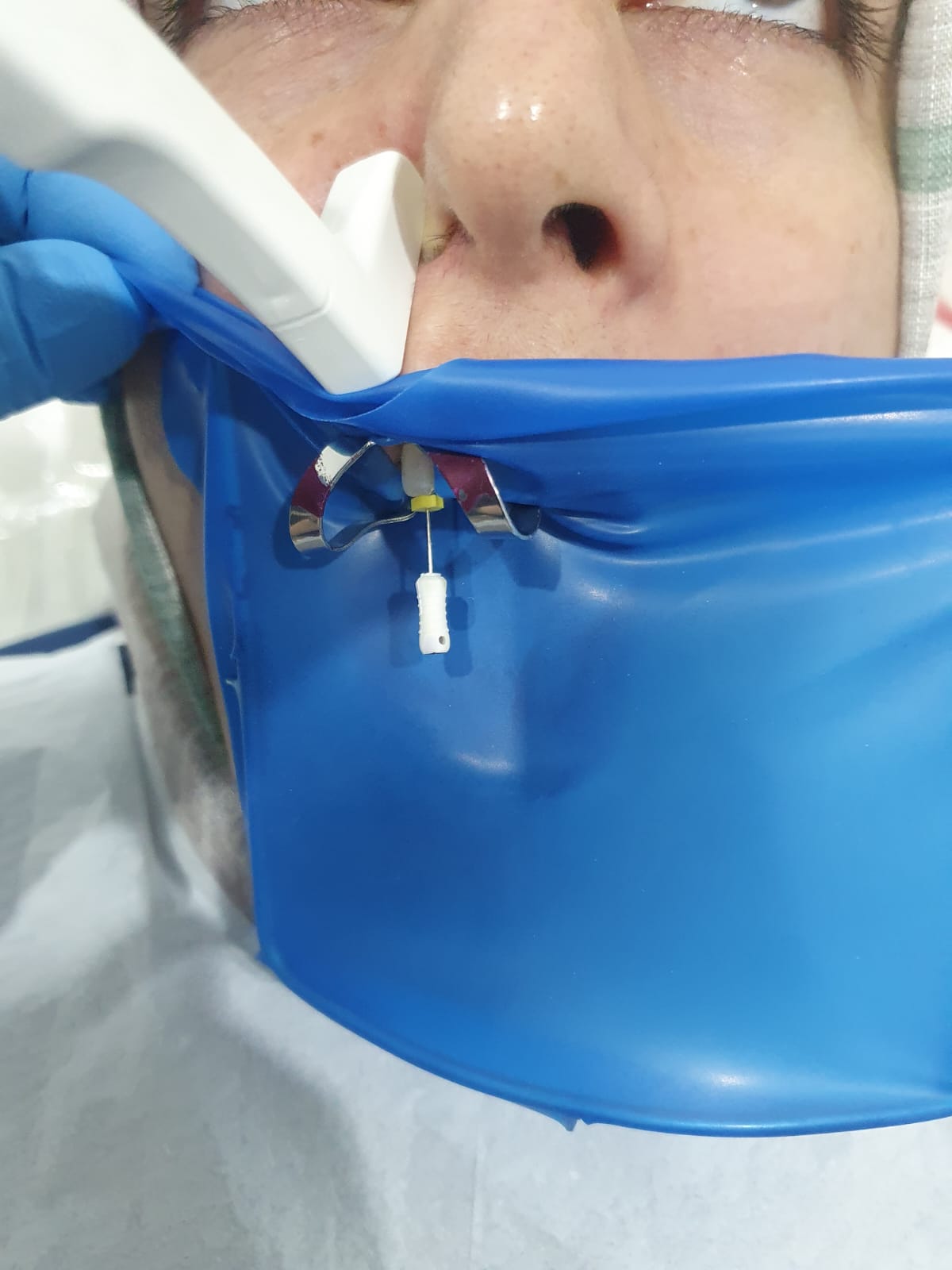

Supplement: Supplementary file 4 — Supplementary Material 4 [file 12903_2024_4562_MOESM4_ESM.jpeg]

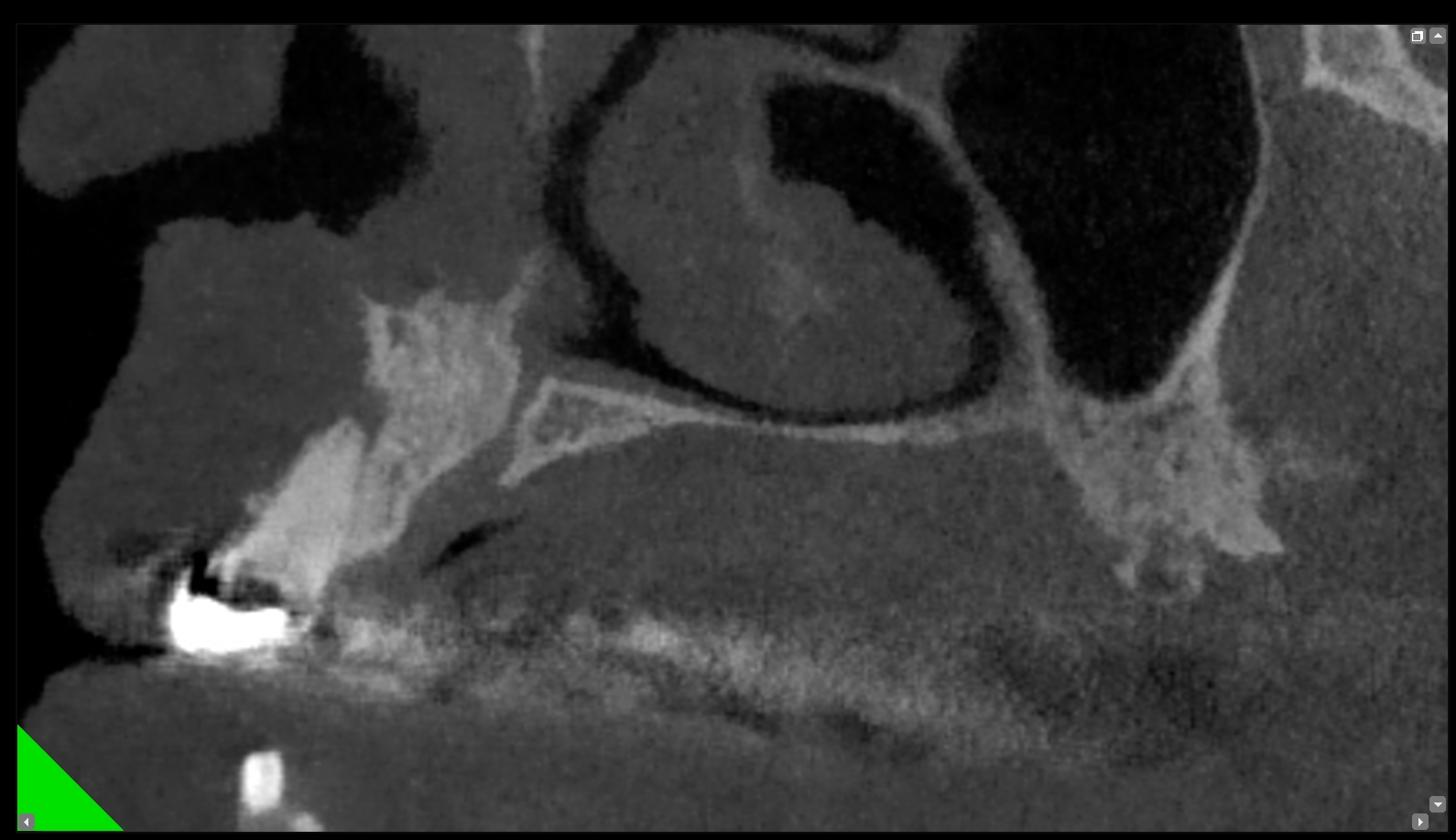

Supplement: Supplementary file 5 — Supplementary Material 5 [file 12903_2024_4562_MOESM5_ESM.jpg]

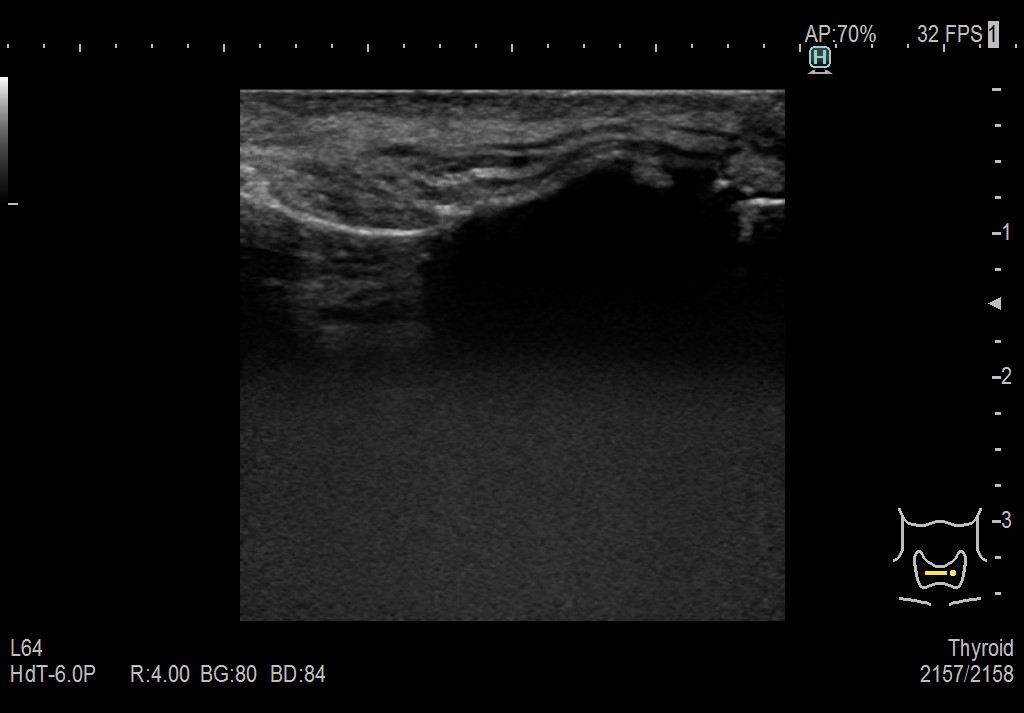

Supplement: Supplementary file 6 — Supplementary Material 6 [file 12903_2024_4562_MOESM6_ESM.jpg]

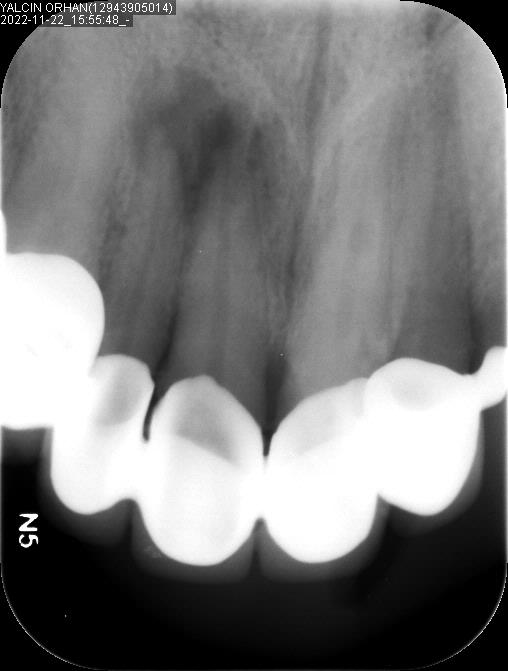

Supplement: Supplementary file 7 — Supplementary Material 7 [file 12903_2024_4562_MOESM7_ESM.jpg]

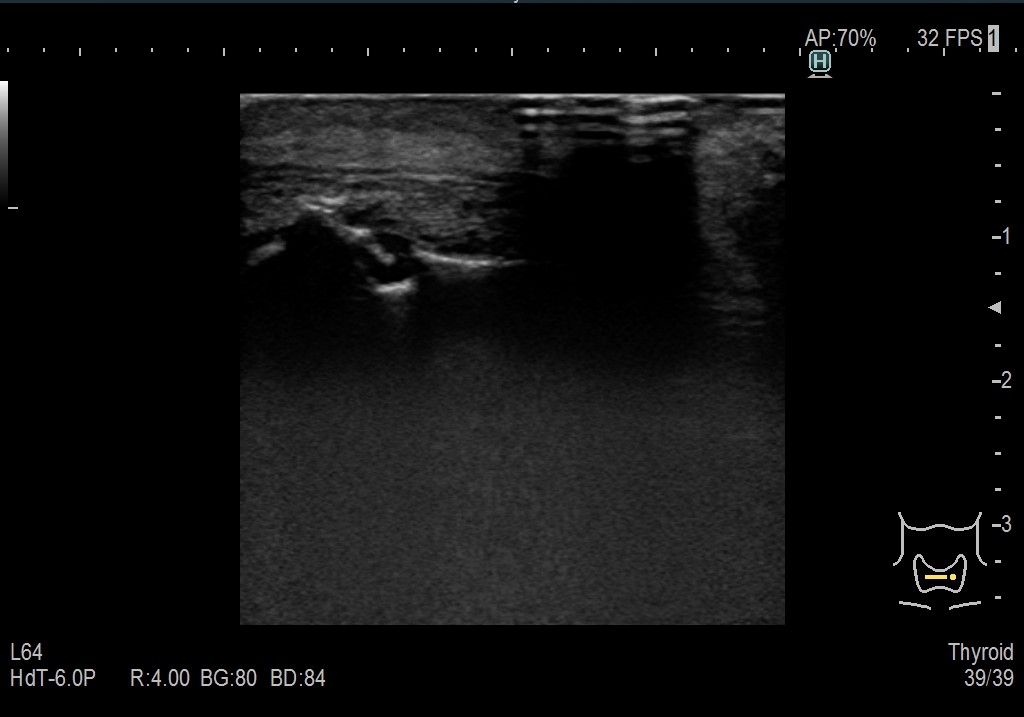

Supplement: Supplementary file 8 — Supplementary Material 8 [file 12903_2024_4562_MOESM8_ESM.jpg]

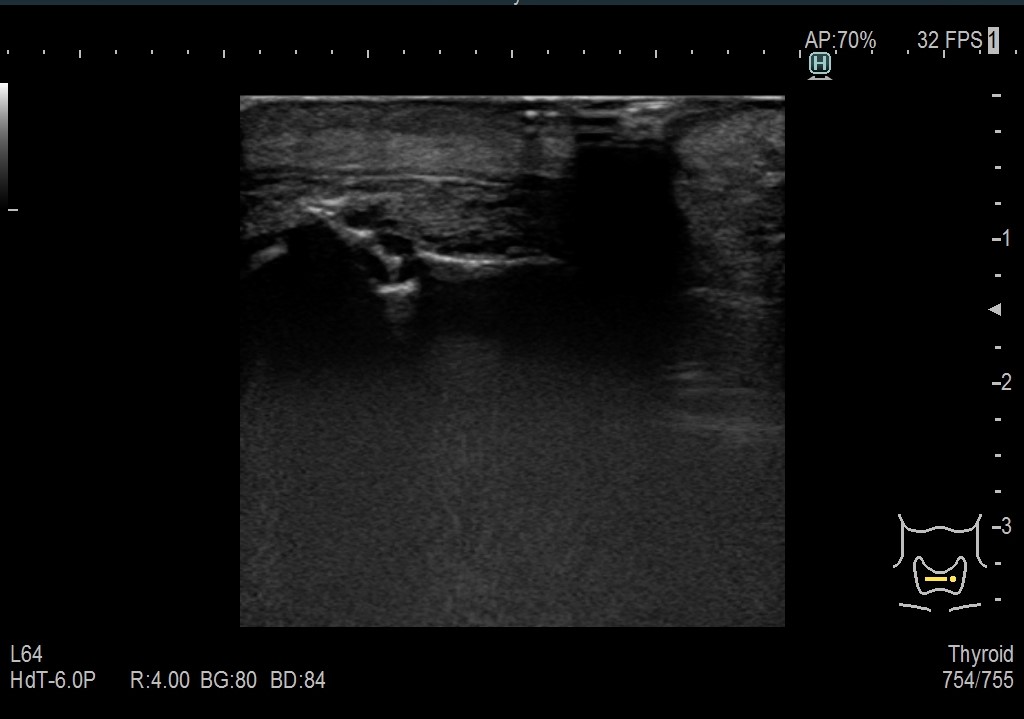

Supplement: Supplementary file 9 — Supplementary Material 9 [file 12903_2024_4562_MOESM9_ESM.jpg]
